# Can Scorpion Venom Peptides Be Safely Used in Cardiovascular Therapy: A Systematic Review

**DOI:** 10.7759/cureus.105747

**Published:** 2026-03-24

**Authors:** Sandeep V Binorkar, Ranjeet Sawant, Rashtrapal N Ukey, Ravindra Bhat

**Affiliations:** 1 Agadatantra (Ayurvedic Toxicology and Forensic Medicine), Government Ayurveda College, Nanded, Nanded, IND; 2 Rasashastra and Bhaishajya Kalpana, K. G. Mittal Ayurvedic College, Mumbai, IND; 3 Kayachikitsa (Ayurvedic Medicine), Karnataka Ayurveda Medical College, Mangalore, IND

**Keywords:** bradykinin-potentiating peptides, cardioprotection, cardiovascular therapy, ion channel modulators, scorpion venom, toxicity

## Abstract

Scorpion venom contains numerous bioactive peptides with potent cardiovascular effects, including bradykinin-potentiating peptides (BPPs), ion channel modulators, and cardioprotective molecules. These peptides show promise for conditions such as hypertension, cardiac injury, and arrhythmias. However, concerns regarding toxicity, immunogenicity, and off-target actions have limited their clinical development. This systematic review evaluates the therapeutic potential and safety of scorpion venom peptides for cardiovascular applications. A systematic search of PubMed, Scopus, Google Scholar, and Semantic Scholar identified 1,141 articles. Screening of 463 abstracts and full-text review of 446 eligible studies resulted in 17 publications meeting the inclusion criteria. Extracted data included mechanisms, efficacy, toxicity, and translational challenges. BPPs consistently demonstrated ACE inhibition, B2 receptor activation, and significant antihypertensive effects in animal models. Several peptides also showed cardioprotective activities by reducing oxidative stress, inflammation, and apoptosis. Ion channel-active peptides influenced cardiac electrophysiology, but many showed proarrhythmic risks due to hERG potassium channel blockade or interactions with Nav1.4/1.5 sodium channels. Structure-activity modification studies improved specificity and reduced toxicity in selected variants. Despite encouraging preclinical data, no scorpion venom peptide has progressed to clinical trials for cardiovascular indications. Major barriers include immunogenicity, instability, delivery challenges, and safety concerns. Scorpion venom peptides represent promising leads for novel cardiovascular therapeutics, particularly as antihypertensive and cardioprotective agents. However, significant toxicological and translational limitations remain. Advances in peptide engineering, targeted delivery, and clinical evaluation are crucial for safely harnessing their therapeutic potential.

## Introduction and background

Scorpion venom, a complex mixture of bioactive molecules, has evolved to efficiently immobilize prey and deter predators. Among its components, peptides have emerged as desirable candidates for drug development due to their high specificity and potency in modulating physiological processes, including those central to cardiovascular health [1-5]. Recent systematic reviews and advances in omics technologies have identified several scorpion venom peptides with direct actions on the cardiovascular system. These include canonical bradykinin-potentiating peptides that inhibit angiotensin-converting enzyme (ACE); non-canonical peptides acting as B2 receptor agonists; and peptides that modulate cardiomyocyte proteins, inotropic activity, and ion channel function, such as hERG potassium channel blockers and sodium current inhibitors in ventricular myocytes [1-3,6].

The therapeutic promise of these peptides lies in their ability to target key mechanisms underlying hypertension, arrhythmias, and other cardiovascular disorders. For example, bradykinin-potentiating peptides can enhance vasodilation and lower blood pressure, while ion channel modulators may correct abnormal cardiac excitability [1-3,6]. The structural and functional diversity of scorpion peptides allows for the development of highly selective drugs, potentially reducing off-target effects compared to conventional small molecules [3-5]. Furthermore, advances in peptide engineering and drug delivery systems are being explored to improve their pharmacokinetic profiles and tissue targeting [3,5,7].

Scorpion venom should be investigated for cardiac ailments because its bioactive peptides exhibit ion channel-modulating, inotropic, and cardioprotective properties that may offer novel mechanistic targets beyond conventional drugs. Current pharmacotherapy for cardiovascular diseases is limited by suboptimal efficacy in refractory cases, adverse effects such as arrhythmia and hypotension, drug resistance, and lack of highly selective ion-channel modulation. Therefore, exploring scorpion venom-derived peptides may help address these therapeutic gaps and provide more targeted treatment options.

However, translating scorpion venom peptides from bench to bedside is fraught with challenges. Chief among these are concerns about toxicity and immunogenicity, as some peptides can disrupt normal ion channel function in non-target tissues, leading to adverse effects such as arrhythmias or neurotoxicity [2,3,7]. The low targeting specificity and high toxicity of specific peptides have historically limited their clinical application, but recent research is making strides in overcoming these barriers through molecular modifications and advanced delivery technologies [3,5,7]. Additionally, the complexity of venom composition and the need for precise dosing further complicate their development as safe therapeutics [3,5,7].

Despite these hurdles, the pharmaceutical landscape is witnessing a growing interest in venom-derived peptides, with some already approved for non-cardiovascular indications and others progressing through preclinical and early clinical trials [5]. The continued identification and characterization of novel peptides, coupled with improvements in synthetic biology and drug delivery, are expected to accelerate the development of safer and more effective cardiovascular therapies [1,3,5]. The present article is an attempt to systematically review the available research on the issue.

## Review

Methods

A systematic review was conducted using sources such as Scopus, Google Scholar, Semantic Scholar, and PubMed.

The search strategy covered studies published from January 1995 to October 31, 2025, reflecting the temporal range of the references included. The last database search was conducted on October 31, 2025, and this will be explicitly stated in the Methods section in the revision.

The following structured Boolean strategy was used (adapted per database syntax): (“scorpion venom” OR “scorpion toxin*” OR “scorpion peptide*” OR “bradykinin-potentiating peptide*”) AND (“cardiovascular” OR “hypertension” OR “cardiac” OR “myocardial” OR “arrhythmia” OR “ion channel” OR “hERG” OR “Nav1.5”) AND (“therapy” OR “therapeutic” OR “treatment” OR “drug development” OR “safety” OR “toxicity”).

PubMed, Scopus, Google Scholar, and Semantic Scholar were the databases searched.

Inclusion criteria

The review included original in vitro, in vivo, translational, or systematic review studies, as well as studies evaluating scorpion venom-derived peptides with cardiovascular relevance. It also included articles reporting on efficacy, mechanisms, safety, toxicity, or translational data, limited to English-language publications published between January 1995 and October 2025.

Exclusion criteria

Studies unrelated to cardiovascular applications were excluded, along with pure toxicology reports lacking therapeutic context. Case reports of envenomation without mechanistic or therapeutic analysis were also excluded, as were conference abstracts without available full text and duplicate publications.

**Figure 1:**
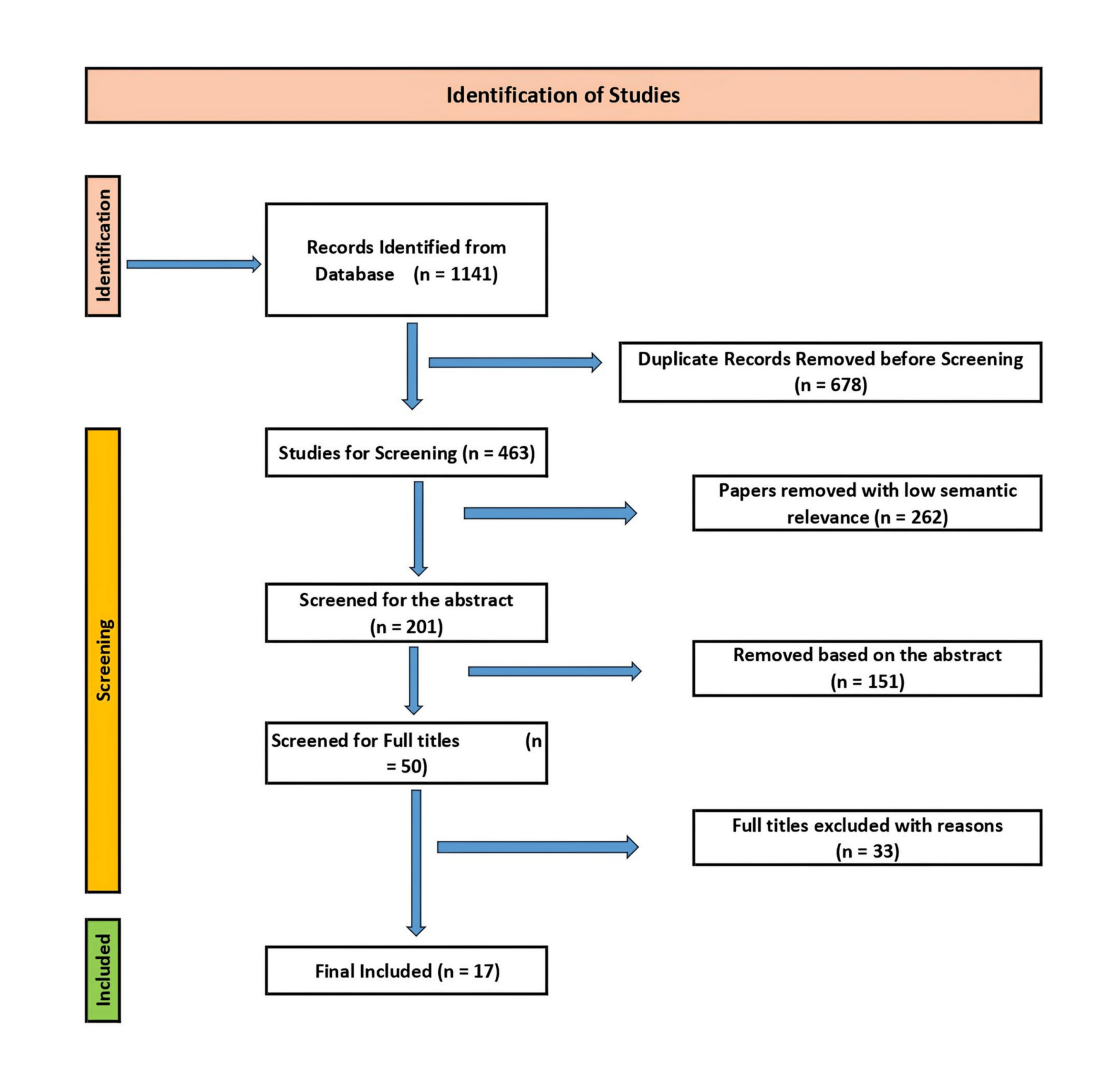
Systematic review flow diagram for scorpion venom peptides in cardiovascular therapy.

Search queries were executed, focusing on mechanisms, safety, toxicity, clinical trials, and translational barriers. The search strategy included terms related to scorpion venom peptides, cardiovascular therapy, safety, toxicity, and clinical translation until October 2025. Of the 1,141 identified papers, 463 were screened, and 17 were included in this review. The search strategy adopted is shown in the PRISMA diagram (Figure 1). 

In this study, a meta-analysis was not performed because the included studies were highly heterogeneous in design (in vitro, animal models, mechanistic studies, and narrative/systematic reviews), peptide types, outcome measures (e.g., blood pressure reduction, electrophysiology parameters, oxidative stress markers), and experimental conditions, precluding meaningful quantitative pooling. Additionally, no randomized controlled human trials were identified, and most preclinical studies lacked standardized effect size reporting, making statistical synthesis inappropriate. Therefore, a qualitative systematic review approach was considered methodologically more appropriate and scientifically justified.

Review

Scorpion venom peptides represent a structurally and functionally diverse class of bioactive molecules with emerging relevance to cardiovascular therapeutics. Among these, bradykinin-potentiating peptides (BPPs) have attracted considerable attention due to their ability to inhibit angiotensin-converting enzyme (ACE) and, in some cases, act as agonists of bradykinin B2 receptors, resulting in significant antihypertensive effects [1,3,8-12]. In parallel, several scorpion venom peptides function as modulators of cardiac ion channels, including voltage-gated sodium channels and potassium channels such as hERG. Through these mechanisms, they exert profound effects on cardiac electrophysiology, myocardial contractility, and vascular tone, highlighting both their therapeutic promise and inherent pharmacological complexity [1,13-16].

In addition to their hemodynamic actions, scorpion venom peptides have demonstrated cardioprotective properties in experimental models. Preclinical studies indicate that selected peptides attenuate oxidative stress, suppress inflammatory signaling pathways, and reduce apoptosis in models of myocardial injury [3,8,9,17]. These pleiotropic effects suggest that venom-derived peptides may offer benefits beyond blood pressure regulation, particularly in pathological states involving ischemia-reperfusion injury, chronic inflammation, and myocardial remodeling.

Evidence from preclinical safety and efficacy studies further supports the cardiovascular potential of these peptides while underscoring critical limitations. In animal models, particularly rodents, BPPs and related peptides have shown efficacy in reducing systemic blood pressure, preserving myocardial structure, and modulating cardiac function [3,8-13]. However, toxicity remains a significant concern, especially for peptides targeting sodium and potassium channels, which may provoke arrhythmias or impair cardiac contractility at higher doses or due to off-target interactions [14-16,18]. To overcome these challenges, extensive structure-activity relationship analyses and mutagenesis studies have been undertaken, yielding optimized peptide variants with enhanced selectivity, reduced toxicity, and improved therapeutic indices [7,17,19].

Despite robust preclinical evidence, clinical translation of scorpion venom peptides for cardiovascular indications remains limited. To date, no scorpion venom-derived peptide has progressed to regulatory approval for cardiovascular therapy, and available human data are minimal [1,2,5,7]. Nevertheless, the successful development of snake venom-derived therapeutics, exemplified by captopril, provides a compelling translational precedent and supports the feasibility of venom-based drug discovery [20-27]. Key barriers to clinical advancement include immunogenicity, limited in vivo stability, challenges in targeted delivery, and off-target toxicity, all of which require systematic resolution before clinical application can be realized [2,7,18,19,26]. Authors and journals that appeared most frequently in the included papers are included in Table 1.

**Table 1:** Key claims and support evidence identified in the papers.

Claim	Evidence Strength	Reasoning	Reference
Scorpion venom peptides have antihypertensive and cardioprotective effects in preclinical models	Strong	Multiple animal studies show efficacy as ACE inhibitors, B2 agonists, and cardioprotective agents	[1,3,8-13]
Some scorpion peptides can cause arrhythmias or cardiac dysfunction	Moderate	Peptides blocking hERG or sodium channels can induce proarrhythmic effects in vitro and in vivo	[14-16,18]
No scorpion venom peptide is currently approved for cardiovascular therapy in humans	Strong	No clinical trials or regulatory approvals to date	[1,2,5,7]
Molecular engineering can reduce toxicity and improve selectivity	Moderate	Mutagenesis and SAR studies have produced safer peptide variants in preclinical models	[7,15,19]
Immunogenicity and delivery remain barriers to clinical translation	Moderate	Animal-derived peptides can provoke immune responses and have stability/delivery challenges	[2,7,26]
Lessons from snake venom drugs suggest a possible path forward	Strong	Captopril and other drugs derived from animal venoms have succeeded in cardiovascular therapy	[20-25]

Safety considerations remain paramount in evaluating scorpion venom peptides as cardiovascular drug candidates. Peptides that block hERG potassium channels or cardiac sodium channels pose a substantial arrhythmogenic risk, with the potential to prolong the QT interval and induce life-threatening cardiac arrhythmias [14-16]. Additionally, insufficient target specificity and systemic toxicity have historically constrained clinical development, although advances in molecular engineering and peptide design are progressively addressing these limitations [2,7,18,19]. Immunogenicity, a common concern with animal-derived peptides, further complicates long-term therapeutic use and necessitates careful evaluation during drug development [2,7,26]. Key claims and supporting evidence identified in the papers and gaps are shown in Tables 2, 3.

**Table 2:** Research coverage by study type and topic; clinical trials are notably lacking.

Study Type / Attribute	Antihypertensive Effects	Cardioprotective Effects	Arrhythmia Risk	Human Clinical Trials	Peptide Engineering
In vitro studies	8	6	7	GAP	5
In vivo animal studies	7	5	6	GAP	3
Clinical trials	GAP	GAP	GAP	GAP	GAP
Systematic reviews/meta- analyses	2	1	1	GAP	1
Mechanistic/molecular studies	6	4	5	GAP	4

**Table 3:** Key open research questions for advancing scorpion venom peptide cardiovascular therapy.

Question	Why
What are the safety and efficacy profiles of scorpion venom peptides in human cardiovascular disease patients?	Clinical trials are needed to translate promising preclinical findings into safe, effective therapies.
How can molecular engineering further reduce the toxicity and improve the selectivity of scorpion peptides?	Optimizing peptide structure may minimize off-target effects and enhance therapeutic potential.
What are the immunogenicity and pharmacokinetic profiles of scorpion venom peptides in humans?	Understanding immune response and stability is critical for safe clinical application.

Most animal studies did not clearly report randomization or allocation concealment (Table 4). Blinding procedures were rarely described. Outcome reporting was generally complete. Overall risk is moderate to high, mainly due to inadequate reporting of bias control measures.

**Table 4:** Risk of bias assessment – animal experimental studies.

Study (First Author, Year)	Random Sequence Generation	Allocation Concealment	Blinding (Performance Bias)	Blinding (Outcome Assessment)	Incomplete Outcome Data	Selective Reporting	Other Bias	Overall Risk
Ahmed 2020 [8]	Unclear	Unclear	High	High	Low	Unclear	Low	Moderate–High
Hasan 2020 [9]	Unclear	Unclear	High	High	Low	Unclear	Low	Moderate–High
Salman 2017 [17]	Unclear	Unclear	High	High	Low	Unclear	Low	Moderate–High
Verano-Braga 2008 [10]	Low	Unclear	Unclear	Unclear	Low	Low	Low	Moderate
Verano-Braga 2010 [11]	Low	Unclear	Unclear	Unclear	Low	Low	Low	Moderate
Gómez-Mendoza 2020 [13]	Low	Unclear	Unclear	Unclear	Low	Low	Low	Moderate

Ion channel and electrophysiology studies show strong experimental rigor (Table 5). There is low detection bias due to objective electrophysiological endpoints, and external validity remains limited (no human clinical translation).

**Table 5:** Risk of bias – in vitro/mechanistic studies.

Study	Experimental Controls	Replicates Reported	Dose–Response Assessment	Statistical Transparency	Overall Methodological Quality
Beltrán-Vidal 2021 [14]	Adequate	Yes	Yes	Clear	High
Xu 2017 [15]	Adequate	Yes	Yes	Clear	High
Zhao 2022 [18]	Adequate	Yes	Yes	Clear	High
Liu 2025 [19]	Adequate	Yes	Yes	Clear	High
De Waard 2020 [16]	Adequate	Yes	Yes	Clear	High

Studies listed in Table 6 show that these are largely narrative or descriptive reviews. The main limitation was the lack of a structured, systematic methodology. Risk of bias is mainly from confounding and selection processes.

**Table 6:** Risk of bias – non-randomized/translational studies.

Study	Confounding	Selection Bias	Classification Bias	Outcome Measurement Bias	Overall Risk
Uzair 2018 [4]	Moderate	Low	Low	Low	Moderate
Baradaran 2023 [5]	Moderate	Low	Low	Low	Moderate
Wiezel 2024 [6]	Moderate	Low	Low	Low	Moderate

Only one review demonstrated high methodological quality (Table 7). The lack of protocol registration and formal bias assessment lowers confidence in others. The 17 included studies in the review are summarized in Table 8.

**Table 7:** Risk of bias – included systematic reviews.

Study	Protocol Registered	Comprehensive Search	Dual Screening	Risk of Bias Assessed	Funding Bias Considered	Overall Quality
Santos 2024 [1]	Unclear	Yes	Unclear	Partial	Unclear	Moderate
Díaz-Gómez 2023 [26]	Yes	Yes	Yes	Yes	Yes	High
Mendes 2023 [2]	Unclear	Yes	Unclear	Partial	Unclear	Moderate

**Table 8:** Summary of 17 included studies in the review.

Author (Year) [Reference]	Study Type	Peptide / Focus	Cardiovascular Target / Outcome	Main Findings
Ahmed (2020) [8]	Animal (Rat)	BPP fraction (Leiurus quinquestriatus)	Doxorubicin cardiotoxicity	Reduced oxidative stress, inflammation, apoptosis; cardioprotection
Hasan (2020) [9]	Animal (Rat)	Bradykinin-potentiating factor	Radiation-induced cardiomyopathy	Improved RAAS modulation; antihypertensive & cardioprotective
Salman (2017) [17]	Animal (Rat)	BPP (L. quinquestriatus)	Blood indices, lipid profile	Improved biochemical markers post-irradiation
Verano-Braga (2008) [10]	Animal / Peptide characterization	Ts Hypotensins	Hypotensive activity	Identified new hypotensin family; BP reduction
Verano-Braga (2010) [11]	Animal / Mechanistic	TsHpt-I	B2 receptor agonism	Confirmed B2 receptor activation; vasodilatory mechanism
Gómez-Mendoza (2020) [13]	Animal / Mechanistic	Ts14 cryptic peptide	Cardiac signaling (AKT/ERK)	Reduced cardiomyocyte contractility; phospholamban modulation
Beltrán-Vidal (2021) [14]	In vitro (Ion channel)	γ-K+ toxin (Centruroides margaritatus)	hERG blockade	Full hERG1 block; arrhythmogenic risk
Xu (2017) [15]	In vitro / Mutagenesis	Buthus martensii peptide mutant	Nav1.4 / Nav1.5	Reduced sodium channel inhibition with retained activity
Zhao (2022) [18]	In vitro / Computational	Analgesic-antitumor peptide	Human Nav1.4/1.5	Identified determinants of sodium channel side effects
Liu (2025) [19]	In vitro / SAR optimization	DKK2 toxin variant	Sodium channel blockade	Improved selectivity; reduced off-target toxicity
De Waard (2020) [16]	In vitro (hiPSC-CMs)	BeKm-1	hERG channel	Confirmed QT-prolongation potential
Uzair (2018) [4]	Translational Review	Multiple venom peptides	Drug development potential	Summarized pharmacological promise
Baradaran (2023) [5]	Translational Review	Therapeutic peptides	Clinical translation	Highlighted peptide-based drug status
Wiezel (2024) [6]	Translational / Descriptive	Tityus spp. toxins	Pharmacological repertoire	Documented venom diversity and potential
Santos (2024) [1]	Systematic Review	Scorpion venom peptides	Cardiovascular diseases	Synthesized antihypertensive/cardioprotective evidence
Díaz-Gómez (2023) [26]	Systematic Review	Synthetic venom-derived peptides	Biomedical applications	High-quality review; included bias assessment
Mendes (2023) [2]	Systematic Review	Ion channel–active peptides	Mechanisms & drug development	Mechanistic insight into channel modulation

Discussion

Scorpion venom peptides represent one of the most structurally diverse and pharmacologically potent groups of naturally occurring biomolecules, and their cardiovascular effects have attracted increased attention over the past decade. The findings of this methodical review highlight both the therapeutic promise and the complex challenges associated with repurposing venom peptides into safe cardiovascular medicines. While the antihypertensive and cardioprotective properties of several bradykinin-potentiating peptides (BPPs) and non-canonical peptides have been well-documented in preclinical studies [1,8-12], their toxicological profiles, unintended pharmacological effects, and inadequate clinical substantiation remain critical limitations.

A crucial observation arising from multiple studies is the dualistic nature of scorpion venom peptides; the exact molecular mechanisms that confer remedial properties can also intervene in toxicity. For illustration, BPPs ply antihypertensive effects substantially by inhibiting ACE or activating B₂ receptors, thereby adding bradykinin bioavailability and enhancing vasodilation [10,11]. These mechanisms are analogous to those exploited in captopril, the classical ACE agent inspired by snake venom factors [21,25,28]. The success of snake venom-derived medicines underscores the implicit translational value of venom peptides. Still, unlike snake venom peptides, scorpion-derived molecules have not yet progressed to clinical evaluation for cardiovascular use [1,2,5,7]. The most significant safety concern linked to this review is the arrhythmogenic eventuality of ion channel-modulating peptides.

Several scorpion poisons widely or incompletely block hERG (KCNH2) potassium channels, which regulate cardiac repolarization [14,16]. hERG channel inhibition is a major red flag in medicine development due to its association with a prolonged QT interval and torsades de pointes. In addition, peptides that interact with voltage-gated sodium channels, including Nav 1.4 and Nav 1.5, may provoke conduction abnormalities or alter myocardial excitability [15,18]. Although mutagenesis and structure-activity relationship (SAR) studies have successfully developed poison variants with reduced sodium channel affinity while retaining salutary bioactivity [15,19], these findings remain confined to in vitro and animal settings. Cardioprotective effects observed in rodent models give another compelling dimension to venom peptide exploration.

Several peptides demonstrate antioxidants, anti-inflammatory, and anti-apoptotic conditioning, reducing doxorubicin-induced cardiotoxicity or radiation-induced cardiac redoing [8,9,17]. These findings indicate a broader remedial window beyond blood pressure regulation, suggesting implicit places in ischemia-reperfusion injury, myocardial inflammation, or heart failure. Nonetheless, the translation of such multimodal effects into human therapy must regard pharmacokinetic challenges, including peptide stability, rapid-fire declination, and limited tissue-specific targeting [2,7].

Another challenging aspect is immunogenicity. As animal-deduced peptides, scorpion poisons may provoke vulnerable responses, including neutralizing antibodies or acute responses [2,27]. Inventions in peptide engineering-such as cyclization, PEGylation, nanoparticle encapsulation, or conjugation with targeting ligands-are being explored to reduce immunogenicity and enhance delivery effectiveness [7,19]. Still, these strategies add complexity to manufacturing and non-supervisory blessings. The absence of clinical trials remains a significant gap. Although peptides deduced from snake venom have successfully reached clinical use for hypertension, thrombosis, and heart failure [20,23,24], scorpion venom peptides remain confined to preclinical exploration. This distinction may stem from the advanced toxicity of scorpion venom, the lower clinical demand for peptide-based cardiovascular medicines, or limited interdisciplinary collaboration among toxinologists, cardiologists, and pharmaceutical chemists. From an unborn exploration perspective, three disciplines crop up as high-precedence areas. First, comprehensive electrophysiological profiling using induced pluripotent stem cell-derived cardiomyocytes (hiPSC-CMs) should become standard practice for assessing cardiotoxicity before the discovery stage [16]. Second, advances in omics technologies and bioinformatics can accelerate the discovery of safer peptide analogs with enhanced selectivity [6]. Third, translational fabrics inspired by the development of poison-derived medicines, including detailed pharmacokinetic modeling, peptide optimization, and phase I safety trials, must be espoused totally. Overall, while the remedial eventuality of scorpion venom peptides in cardiovascular drugs remains substantial, their safe clinical operation will bear rigorous evaluation, innovative engineering, and multidisciplinary collaboration. Venom peptides continue to provide a unique molecular pulpit that does not present in conventional medicine libraries, and with strategic scientific investment, they may ultimately expand the arsenal of cardiovascular correctors. Key open research questions for advancing scorpion venom peptide cardiovascular therapy are shown in Table 4.

Historically, many animal experimental studies did not consistently emphasize methodological reporting elements such as randomization, allocation concealment, and blinding, and these criteria were not always strictly required in earlier publication standards. However, the intention in highlighting these aspects was not to criticize the included studies but to report observations from the structured risk-of-bias assessment conducted using the SYRCLE framework, which evaluates internal validity parameters analogous to those used in clinical research. The absence of explicit reporting does not necessarily indicate that these procedures were not performed, but rather that they were not clearly documented in the original publications. Reporting standards in animal research have evolved over time, and guidelines such as ARRIVE now encourage more transparent reporting of randomization, blinding, and allocation procedures.

In consonance with the aim of the manuscript, which is to critically discuss the therapeutic potential and safety of scorpion venom peptides in the therapy of cardiovascular diseases, it is equally important to discuss the arguments against the evaluation of scorpion venom peptides in the therapy of heart diseases. It is an established fact that scorpion venom is cardiotoxic in nature and has the potential for the induction of life-threatening arrhythmias, QT interval prolongation, autonomic storm, and cardiovascular instability by modulating the hERG potassium channels and the sodium channels. Considering the high morbidity and mortality associated with cardiovascular diseases, the introduction of biologically active toxins would not be safe and would pose a significant risk to the patient population. Therefore, while scorpion venom peptides provide mechanistic insights and drug discovery leads, their direct therapeutic development for cardiac diseases must be approached with extreme caution and strong preclinical justification.

A structured methodological quality appraisal was conducted to assess the threat of bias and methodological quality evaluation for all included studies using standardized and study-type-applicable tools. Animal experimental studies were assessed using the SYRCLE tool for threat of bias (adapted from the Cochrane tool for threat of bias framework); non-randomized and translational examinations were estimated using ROBINS-I principles, and included methodical reviews were rated using AMSTAR 2. In vitro mechanistic and electrophysiological studies were assessed using internal validity criteria focusing on experimental controls, reproducibility, and statistical translucency.

In consonance with the aim of the manuscript, which is to critically discuss the therapeutic potential and safety of scorpion venom peptides in the therapy of cardiovascular diseases, it is equally important to discuss the arguments against the evaluation of scorpion venom peptides in the therapy of heart diseases. It is an established fact that scorpion venom is cardiotoxic in nature and has the potential for the induction of life-threatening arrhythmias, QT interval prolongation, autonomic storm, and cardiovascular instability by modulating the hERG potassium channels and the sodium channels. Considering the high morbidity and mortality associated with cardiovascular diseases, the introduction of biologically active toxins would not be safe and would pose a significant risk to the patient population. Therefore, while scorpion venom peptides provide mechanistic insights and drug discovery leads, their direct therapeutic development for cardiac diseases must be approached with extreme caution and strong preclinical justification.

The utmost of the in vivo examinations assessing anti-hypertensive, cardioprotective, or electrophysiological effects of scorpion venom peptides were conducted in rodent models. While these studies constantly demonstrated natural efficacy, methodological limitations were constantly observed.

The primary sources of bias included random sequence generation and allocation concealment. These were infrequently described explicitly. Although experimental grouping was reported, formal randomization procedures were generally not detailed, resulting in an unclear threat of selection bias. Bedazzling blinding of investigators during intervention administration (performance bias) and outgrowth assessment (discovery bias) was infrequently reported. This represents a significant methodological concern, particularly for endpoints similar to histopathology, biochemical assays, and functional cardiac assessments. Deficient outgrowth data waste rates were generally low, and most studies appeared to report complete datasets. Still, formal waste analyses were infrequently handled. Picky reporting of utmost studies reported anticipated biochemical and functional endpoints, but the absence of preregistered protocols limited assessment of reporting bias.

In vitro examinations, including ion channel assays, patch-clamp studies, and molecular commerce analyses, demonstrated comparatively strong methodological rigor. Because electrophysiological issues are objective and instrument-grounded, discovery bias is innately low. Still, these studies are limited by the indirectness of substantiation, as cellular models do not completely replicate mortal cardiovascular physiology. Likewise, numerous assays were conducted in heterologous expression systems or isolated cardiomyocytes, which may not capture systemic pharmacokinetic or immunogenic properties. Therefore, the internal validity of these studies was considered high, but external validity and translational connection remain limited.

Non-randomized and translational studies show that narrative and translational reviews were estimated using ROBINS-I-aligned principles. These studies generally synthesized mechanistic and pharmacological data but did not employ formal methodology. Common methodological enterprises included a lack of predefined addition criteria, absence of structured threat of bias assessment, implicit for selection bias in study addition, and limited discussion of confounding factors, similar to cure variability, peptide variations, or interspecies differences. These studies were thus classified as having a moderate threat of bias, primarily due to methodological diversity and non-systematic selection processes.

Apart from this, the methodological quality of preliminary methodological reviews varied. Using AMSTAR 2 criteria, comprehensive literature searches were generally performed. Still, protocol enrollment was inconsistently reported. The binary independent webbing and formal threat-of-bias assessments were not always easily described. Backing source bias and conflict-of-interest analysis were inconsistently addressed. And only one review met criteria harmonious with high methodological quality. Others were rated as moderate due to deficient translucency in methodology.

A critical finding of this review is the complete absence of randomized controlled clinical trials assessing scorpion venom peptides for cardiovascular indications. Accordingly, there's no direct mortal substantiation regarding safety, tolerability, optimal dosing, or long-term cardiovascular issues. Certainty of substantiation for clinical operation remains very low. All conclusions are deduced from circular preclinical models. This represents the most significant limitation in the current substantiation base.

From a GRADE perspective, the overall certainty of evidence varies across outcomes. The antihypertensive effect of scorpion venom peptides is supported by consistent animal data demonstrating ACE inhibition and B2 receptor activation, resulting in moderate certainty, although the evidence remains indirect and preclinical. Cardioprotective effects, including attenuation of oxidative stress, inflammation, and apoptosis, are supported by experimental studies but are limited by methodological concerns in animal models, leading to low to moderate certainty. The evidence regarding arrhythmogenic risk, particularly related to hERG potassium channel blockade and Nav1.5 sodium channel interactions observed in electrophysiological studies, is mechanistically consistent and therefore graded as moderate certainty. In contrast, certainty regarding human therapeutic efficacy is very low, as no randomized clinical trials have been conducted. Overall, the certainty of evidence was downgraded due to risk of bias in animal experiments, indirectness arising from reliance on non-human models, and imprecision related to translational applicability to clinical cardiovascular practice.

While preclinical findings on scorpion venom peptides are biologically compelling and mechanistically coherent, methodological limitations in animal studies and the absence of human clinical trials substantially limit translational confidence. The reported antihypertensive and cardioprotective effects-mediated through pathways such as ACE inhibition, B2 receptor activation, and ion channel modulation-are supported by reproducible experimental evidence; however, significant safety concerns, particularly hERG channel blockade and cardiac sodium channel inhibition, necessitate rigorous electrophysiological screening before clinical advancement. Strengthening the evidence base will require well-designed, randomized, and blinded animal studies adhering to ARRIVE guidelines, comprehensive preclinical electrophysiological evaluation using human-induced pluripotent stem cell-derived cardiomyocytes, formal toxicokinetic and immunogenicity profiling, and ultimately, carefully conducted Phase I safety trials following peptide optimization.

The emerging approaches in peptide discovery and optimization, including high-resolution proteomic identification of venom peptides and computational or structure-guided peptide design strategies, are accelerating the discovery and refinement of therapeutic peptide candidates. These developments strengthen the translational framework for venom-derived therapeutics.

Limitations of the study

In the present study, a meta-analysis was not performed because the included studies were highly heterogeneous in design. Additionally, no randomized controlled human trials were identified, and most preclinical studies lacked standardized effect size reporting, making statistical synthesis inappropriate. Therefore, a qualitative systematic review approach was considered methodologically more appropriate and scientifically justified. Also, the review protocol was not prospectively registered and hence acknowledged as a methodological limitation. 

## Conclusions

Scorpion venom peptides show significant promise for cardiovascular therapy, particularly as antihypertensive and cardioprotective agents and as a largely untapped resource for cardiovascular drug discovery. However, their safe use in humans has not yet been established due to concerns about toxicity, specificity, and limited clinical data. Further research, including rigorous preclinical and clinical trials and optimization of peptide properties, is needed before these molecules can be safely integrated into cardiovascular medicine.
